# Detecting Bipolar Depression From Geographic Location Data

**DOI:** 10.1109/TBME.2016.2611862

**Published:** 2016-10-25

**Authors:** N. Palmius, A. Tsanas, K. E. A. Saunders, A. C. Bilderbeck, J. R. Geddes, G. M. Goodwin, M. De Vos

**Affiliations:** Department of Engineering Science, Institute of Biomedical Engineering, University of Oxford; Department of Engineering Science, Institute of Biomedical Engineering, and also with the Oxford Centre for Industrial and Applied Mathematics, Mathematical Institute and the Nuffield Department of Clinical Neurosciences, Sleep and Circadian Neuroscience Institute, University of Oxford; Department of Psychiatry, University of Oxford; Department of Engineering Science, Institute of Biomedical Engineering, and also with the Nuffield Department of Clinical Neurosciences, Sleep and Circadian Neuroscience Institute, University of Oxford, Oxford OX3 7DQ, U.K.

**Keywords:** Bipolar disorder, classification, community study, depression, geolocation

## Abstract

**Objective:**

This paper aims to identify periods of depression using geolocation movements recorded from mobile phones in a prospective community study of individuals with bipolar disorder (BD).

**Methods:**

Anonymized geographic location recordings from 22 BD participants and 14 healthy controls (HC) were collected over 3 months. Participants reported their depressive symptomatology using a weekly questionnaire (QIDS-SR_16_). Recorded location data were preprocessed by detecting and removing imprecise data points and features were extracted to assess the level and regularity of geographic movements of the participant. A subset of features were selected using a wrapper feature selection method and presented to 1) a linear regression model and a quadratic generalized linear model with a logistic link function for questionnaire score estimation; and 2) a quadratic discriminant analysis classifier for depression detection in BD participants based on their questionnaire responses.

**Results:**

HC participants did not report depressive symptoms and their features showed similar distributions to nondepressed BD participants. Questionnaire score estimation using geolocation-derived features from BD participants demonstrated an optimal mean absolute error rate of 3.73, while depression detection demonstrated an optimal (median ± IQR) F_1_ score of 0.857 ± 0.022 using five features (classification accuracy: 0.849 ± 0.016; sensitivity: 0.839 ± 0.014; specificity: 0.872 ± 0.047).

**Conclusion:**

These results demonstrate a strong link between geographic movements and depression in bipolar disorder.

**Significance:**

To our knowledge, this is the first community study of passively recorded objective markers of depression in bipolar disorder of this scale. The techniques could help individuals monitor their depression and enable healthcare providers to detect those in need of care or treatment.

## I Introduction

Bipolar disorder is a common mental disorder estimated to affect 2–4% of the general population [1]–[4]. Standard descriptions characterise the disorder as one of periods of elation (mania) and depression interspersed with periods of stability (euthymia) [5]. In reality, the clinical picture is far more complex. Mood instability [6], [7] and subsyndromal symptoms persist in euthymia [8], [9], which have a significant impact on functional outcomes for patients [7]. Depressive episodes are both longer in duration [10] and more common [1] than manic episodes. Individuals with bipolar disorder can spend up to a third of their lives suffering with syndromal or subsyndromal depressive symptoms [8]. Depression is the leading cause of functional impairment in bipolar disorder [11] and contributes to the elevated mortality rates in this patient group [12]. Bipolar depression is challenging to treat because of the absence of specific treatments [13] and the risk of precipitating a mixed or manic episode [14], [15].

Early identification and intervention for emergent mood episodes is a key aspect of managing the disorder. Educating patients to identify early symptoms of relapse has been shown to be associated with clinical improvements in time to first manic relapse and social functioning [16]. The ubiquity of mobile technologies has facilitated developments in longitudinal monitoring of mood states in bipolar disorder [17]–[21]. Early initiatives focused on regular self-reporting by patients [17]–[19] but such approaches rely on the engagement of individuals to actively participate, something that may become impaired as an individual becomes more unwell. There is increasing interest in the use of passive data streams that require no active user input in the monitoring of mood states, such as activity levels [20]; phone use [20], [21]; or physiological measurements [22]. Measurable differences in activity levels in bipolar disorder, especially during mania, have been shown in both mouse and controlled human studies [23]. Translating this to a wider scale, a number of features of bipolar depression have behavioral manifestations that may be detectable using passive data streams from mobile devices. For example, anhedonia, poor concentration, and low energy may all be manifest in changes in geographic movements such as spending less time away from home, visiting fewer places or disruption to daily routines.

Grünerbl *et al.* [20], as part of the wider MONARCA project, explored the relationship between clinician-rated mood states and movement using a combination of accelerometry and global positioning system (GPS) traces in a small cohort of hospitalized patients with bipolar disorder. Classification based on these features varied between participants with a mean of 81%. However, movement was restricted to some extent because of the hospitalized nature of the cohort.

In a community cohort of individuals recruited over the internet, Saeb *et al.* [21] reported classification accuracy of 86.5% for detecting mild depression as measured using the Patient Health Questionnaire-9 (PHQ-9) [24]. The participants in this sample were not diagnosed with any mental disorder and as such the generalizability of these findings is unclear.

In this study, we aim to detect clinically significant levels of depression using features extracted from geographic location recordings in a community cohort of bipolar patients.

## II Methods

### A Data Collection

Data for this study were collected from 49 participants between May and July 2015 in the ongoing Automated Monitoring of Symptom Severity (AMoSS) study^1^ at the University of Oxford [25]. The AMoSS study uses a custom open source smartphone app to collect behavioral data from participants and self-reported clinically validated questionnaires to collect psychiatric state. Participants in the study are either healthy controls (HC) without symptoms of any mental disorder or patients diagnosed with bipolar disorder (BD), or borderline personality disorder (BPD). In this study, we focus exclusively on BD and HC participants while our findings from BPD participants will be reported in a follow-up study. Participants were recruited from the area around Oxford, U.K. for an initial three-month period, with an option to remain in the study for 12 months. Many of the BD participants were recruited from previous studies and the remainder were recruited through local community advertising and word-of-mouth. All participants were screened by an experienced psychiatrist using the Structured Clinical Interview for DSM IV.

Location data were collected from 20 HC and 29 BD participants. The recorded data were split into calendar-week epochs and manually inspected and labeled as described later. This resulted in 310 weeks of labeled data for analysis where there were sufficient data available to calculate features (124 HC; 186 BD) from 36 participants (14 HC; 22 BD). Table I shows demographic data for these participants.

Participants were asked to complete the self-report 16-item quick inventory of depressive symptomatology (QIDS-SR_16_) questionnaire [26] on a weekly basis through the True Colours monitoring system [18], [19]. The QIDS-SR_16_ questionnaire is a 16 question diagnostic and monitoring tool for depression covering all nine DSM-IV symptom criterion domains of: sad mood, concentration, self-criticism, suicidal ideation, loss of interest, energy/fatigue, sleep disturbance, changes in appetite/weight, and psychomotor agitation/retardation. QIDS-SR_16_ returns a score between 0 (no symptoms) and 27 (severe symptoms), with defined thresholds [26] of no depression (0–5); mild depression (6–10); moderate depression (11–15); and severe depression (≥ 16). Participants were prompted to complete the questionnaire through a weekly email sent at a time convenient to them. The questionnaire could be completed at any time before the next weekly prompt, and the value was recorded at the time of their response.

Because participants may forget, or be unable, to respond, each calendar week of data was labeled using either a response within 3.5 d either side of the week if there was only one such response; or the mean of the linear interpolation between all responses within 7 d either side of the week, calculated for each day during the week.

**Fig. 1:**
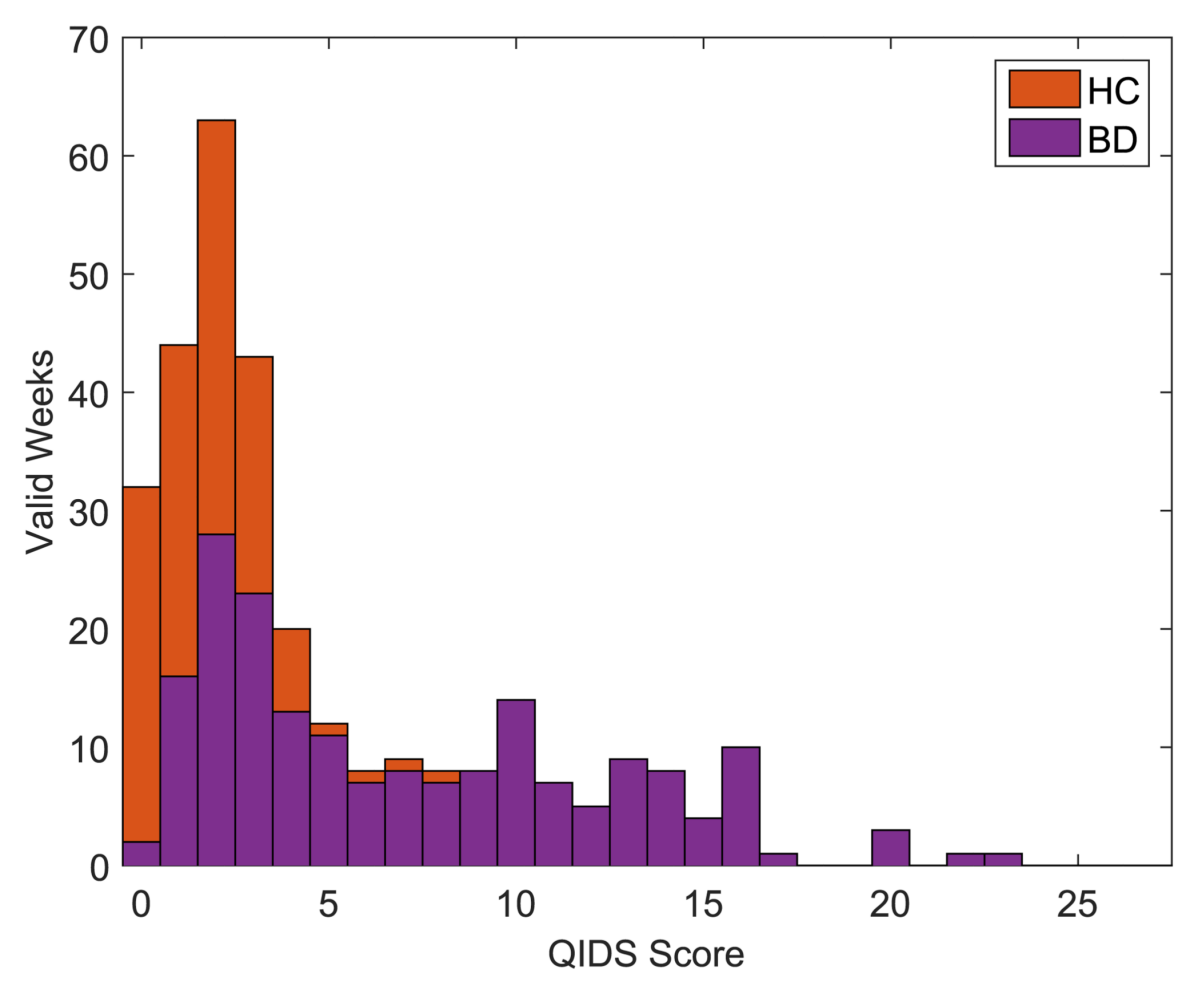
Stacked QIDS score distribution in labeled weeks. The height of each bar shows the total number of weeks labeled and the shading indicates how many are from each group.

QIDS scores for labeled weeks were distributed as shown in Fig. 1. Of the 186 labeled weeks from 22 BD participants, 43 weeks (23.1%) from seven participants had a QIDS score ≥ 11 (the accepted clinical threshold of moderate depression). None of the HC participants had a QIDS score ≥ 11, therefore, analysis was only performed on the BD participants.

Participants were also provided with an Android-based Samsung GALAXY S III or S4 smartphone with a custom app installed that records the anonymized geographic location of the phone. The data were anonymized relative to a random location on earth to protect the participants’ privacy.

### B Data Preprocessing

Location data recorded on Android is a fusion of different location data sources including GPS, nearby wireless network access points and triangulation of the distance to any nearby cellphone towers [27], [28], each of which have different advantages and disadvantages in terms of availability, accuracy and power requirements. When requesting the current location from the Android operating system, the location returned can originate from any of the above sources depending on the resource availability on the device at the time.

**Fig. 2:**
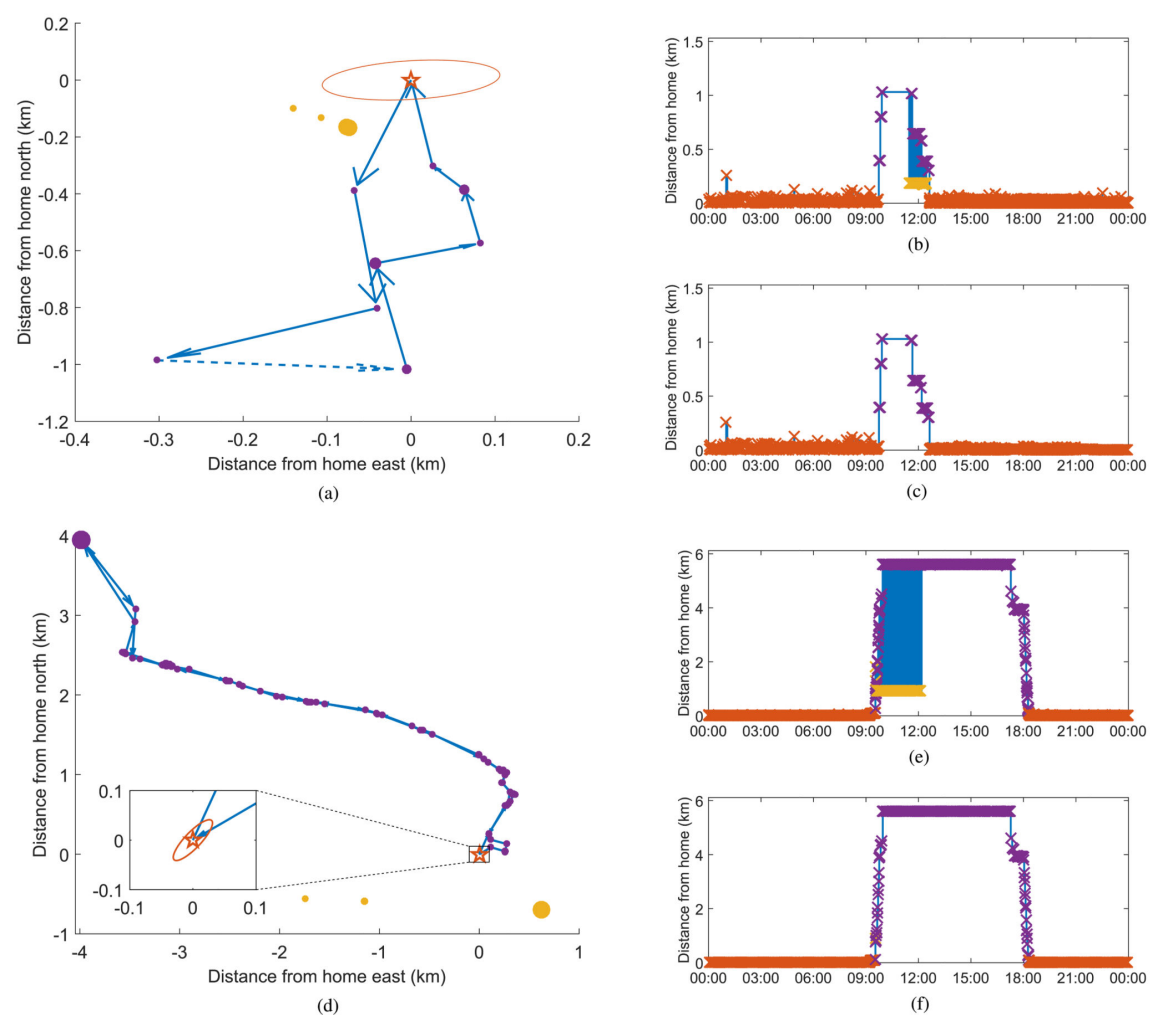
Filtering of inaccurate data for a typical weekend [top, (a)–(c)] and weekday [bottom, (d)–(f)] day from different participants. Plots (a) and (b) on the left show the coordinates where the participant was recorded relative to their home location. The dark orange star represents (0, 0), which is assumed to be the home location of the participant from which other distances are computed. The surrounding ellipse shows the 95% confidence interval of the points recorded at the assumed home location modeled as a multivariate normal distribution. The purple points are accurate location readings with the path between them joined by the blue arrows. The broken arrow in (B) indicates that there is a gap of longer than 15 min where no data were recorded between the two points and therefore the location of the participant cannot be accurately determined. The yellow points are inaccurate noise recorded at the same time as the accurate recordings. Graphs (b) and (c) and (e) and (f) on the right show the Euclidean distance from where the participant is recorded to their assumed home location over the duration of the day. Plots (b) and (e) show the original unfiltered data with noise, and plots (c) and (f) show the filtered data. The colors of the markers in graphs (b) and (c) and (e) and (f) correspond to the locations with the same color shown in plots (a) and (d). The blue shaded areas in (b) and (e) are showing rapid transitions between the purple (accurate) and yellow (inaccurate) locations (these transitions were excluded from plots (a) and (d) for clarity). It can clearly be seen that the yellow points are inaccurate because they are located far from what appears to be a reasonable path in the plots on the left and that they occur concurrently with the locations in the reasonable path in the graphs on the right. The size of the point in the plots on the left indicates the length of time spent in each location, scaled between 10 min and 1 h. Plots (c) and (f) show the preprocessed data traces used for further analysis.

In practice, it was found that the Android operating system would often provide inaccurate location values interspersed between what would appear to be more accurate location values. The inaccurate location values were often provided at a higher sample rate (around 350 samples per hour rather than the more usual 250). This is demonstrated in the sample data from two participants shown in Fig. 2(a) and (d). These graphs show location coordinates recorded from participants over a weekend and weekday day and the route that they most likely took. The dark orange star indicates the assumed home location of the participant (determined as described in subsection II.C.2, Clustering Stationary Locations below) and the surrounding ellipse represents the 95% confidence interval of the data points recorded at this location modeled as a multivariate normal distribution. The three clusters south-west of the ellipse in Fig. 2(a) and south of the home location in Fig. 2(d) are samples that appear to be of low accuracy. This can be seen more clearly in Fig. 2(b) and (e), where the vertical axis is now the Euclidean distance from home to each location sample and the horizontal axis is time. This provides a convenient way to visualize the change in location over time.

Many participants had sections of missing data in the locations recorded, perhaps because the phone was switched off (either intentionally or because the battery was depleted) or because the phone was unable to obtain a GPS location or connect to the Internet to process data. This has also been found in previous studies. For example, Saeb *et al.* [21] excluded over 50% of participants (22 out of 40) from their analysis of GPS locations due to insufficient data and Grünerbl *et al.* [20] reported that of the daily location data recorded for each of their 12 participants, a mean of 31% of days recorded from each participant were insufficient for analysis.

To reduce the effects of these data issues, the preprocessing described below was developed.

#### 1) Filtering

The recorded data were filtered to extract the true paths shown in Fig. 2(c) and (f).

In order to filter the recorded data *D*, it was observed that the low-accuracy data values commonly have exactly the same location coordinates, and are geographically located far from the high-accuracy data points while being temporally close, thus resulting in a high $\frac{d}{dt}D.$ Data were, therefore, filtered by detecting data points that had $\frac{d}{dt}D>100\;\text{km}\;{\text{h}}^{-1},$ either from the previous point, or to the next point. Where there were unique locations that had many such points detected, they were removed from the dataset.

#### 2) Data Down Sampling

The unevenly sampled filtered data were down sampled to a sample rate of 12 samples per hour using a median filter to remove any remaining spurious values. The whole data stream was split into 1 h epochs. If the standard deviation of the recorded data in both latitude and longitude within an epoch was less than 0.01 km, then all samples within the hour were set to the mean value of the recorded data, otherwise a 5 min median filter window was applied to the recorded latitude and longitude in the epoch.

#### 3) Data Imputation

Missing data were imputed in sections where the participant was recorded at a location within 500 m either end of the missing section, and where the missing section had a length of 1) 2 h or less any time; or 2) 12 h or less after 9 pm. The missing section was filled with the mean latitude and longitude of the coordinates on either end.

### C Extracting Location Clusters

Many of the features described in the following rely on extracting the distinct locations where the participant was spending time.

#### 1) Extracting Stationary Locations

The filtered location points were split into stationary locations and transitioning points from the differential of the data. 10 min moving averages were calculated for each sample at time *t* as follows:

(1) $$\begin{array}{cc} {\bar{x}}_{t}^{\text{cen}}=\frac{1}{\left|\tau \right|}\sum_{\tau }\frac{d}{d\tau }D\left(\tau \right) & t-5\text{m}\leq \tau \leq t+5\text{m} \end{array}$$

(2) $$\begin{array}{cc} {\bar{x}}_{t}^{\text{back}}=\frac{1}{\left|\tau \right|}\sum_{\tau }\frac{d}{d\tau }D\left(\tau \right)\; & t-10\text{m}\leq \tau \leq t \end{array}$$

(3) $$\begin{array}{cc} {\bar{x}}_{t}^{\text{fwd}}=\frac{1}{\left|\tau \right|}\sum_{\tau }\frac{d}{d\tau }D\left(\tau \right) & t\leq \tau \leq t+10\text{m}. \end{array}$$

A threshold of *d* was applied where the location at time *t* was considered to be a transitioning location if

(4) $$\left({\bar{x}}_{t}^{\text{cen}}>d\right)\vee \left({\bar{x}}_{t}^{\text{back}}>d\right)\vee \left({\bar{x}}_{t}^{\text{fwd}}>d\right).$$

For this study, a threshold of *d* = 1.5 km h^−1^ was applied.

#### 2) Clustering Stationary Locations

The stationary locations extracted above were clustered to determine the unique locations visited by an individual. This utilized an adaption of the common *K*-means clustering algorithm called *K*-means++ [29]. *K*-means attempts to extract *K* optimal cluster centers from multidimensional data. *K*-means++ improves the performance of *K*-means by choosing the initial cluster centers to be evenly distributed throughout the training dataset. Because the expected number of clusters, *K*, is unknown for the location data used here, the same approach as Saeb *et al.* [21] was used where *K* is set to 1 and incremented while the Euclidean distance between all the clusters remains above a threshold distance *l* apart. For this study, a threshold of *l* = 400 m was used.

The assumed home location was determined by calculating the mode location recorded between 2 am and 7 am for each day *j*, denoted *ĥ*_*j*_ in vector **ĥ**. The global home location was calculated as *ĥ*_*g*_ = mode(**ĥ**) and all the data for each participant was subtracted by *ĥ*_*g*_ to leave location coordinates (0, 0) as the assumed home location.

### D Feature Extraction

Features were extracted from the preprocessed location data as described in Table II.

The location variance; home stay; transition time; total distance; and number of clusters features provide an indication of the overall behavior of participants, focusing on factors around how active the person is.

The entropy (ENT) and normalized entropy (NENT) features are based on information-theoretical entropy [30], which has been proposed as a measure of signal variability [31] and was used by Saeb *et al.* [21] and de Montjoye *et al.* [32] to indicate variability in the time that participants spend in different locations. As an entropy measure, the maximum value of ENT is where the data are least predictable, i.e., where the proportion of time spent in each of the *N* locations is equal. This is counter-intuitive because it would be indicative of a regular daily routine, but from an information theory perspective, the location at each time *t* would be a random draw from an *N*-dimensional categorical distribution with equal probabilities, i.e., the least predictable. In this case, ENT = log *N*, which means that the entropy is highly correlated with the number of clusters recorded. The NENT feature reduces this correlation by scaling the feature value to the range [0*,* 1].

Additionally, regularity in daily routine has long been indicated as a factor in depression [33] and BD [34], [35] where circadian^2^ rhythm disruption is considered by some to be a “core feature” [37]. The three diurnal movement features (DM, DMN, and DMD), based on the feature developed by Saeb *et al.* [21] provide a measure of regularity using the Lomb–Scargle periodogram [38], [39] frequency transform to get the power in the frequencies with wavelengths around 24 h. The Lomb–Scargle periodogram fits sinusoids to data using a least squares fit, which has advantages over the usual fast Fourier transform because it enables extraction of frequency information from unevenly sampled data, and because it does not limit the transform to specific frequencies. The power spectral density of the signal in selected frequencies with wavelengths between 23.5 and 24.5 h was used to calculate the DM features. The Lomb–Scargle periodogram is not affected by DC offset in the signal, but assumes stationarity and is severely affected by the magnitude of the signal.

The basic DM feature can be highly affected by outliers and by the distance travelled from home. For example, an individual with a highly regular diurnal pattern but only travelling a short distance from home would appear to have a low DM, while someone who spends most of their time at home but with one day spent very far from home will have a large DM due to the least-squares fit overcompensating for the outlier day. The diurnal movement on normalized coordinates (DMN) feature attempts to counteract this by using a normalized set of coordinates, where the latitude and longitude are both scaled to have zero mean and unit variance.

Both the aforementioned DM and DMN features are also influenced by the sum of energy in latitude and longitude, which may hide important subtleties in the data. For example, consider an individual with a regular routine, but who works in a job that requires travelling to many different locations, often in different directions from home. The differences in direction mean that the power in either latitude or longitude will be low, even though there is clear regularity in routine. Similarly, consider two individuals who both regularly travel the same distance from home each day for the same duration and at the same times, one travelling directly north, and one travelling north-east. The two individuals will show very different values in the DM and DMN features due to the sum of the power in latitude and longitude. The diurnal movement on the distance from home (DMD) feature was developed to counteract this by operating on the Euclidean distance from home, normalized to have zero mean and unit variance, rather than latitude and longitude. In many cases, this will give a more accurate representation of diurnal movement because the time and distance is more important than the direction.

### E Feature Calculation on Data Subsets

Calculating features over the whole dataset being analyzed, i.e., single calendar weeks in the results presented here, may not accurately represent the characteristics of the individual. For example, someone who works from Monday to Friday will likely exhibit very different feature values during the week and at weekends. For this reason, the features were all calculated over several data subsets.

#### 1) Base Subset

The base subset calculates the features over the whole week being analyzed.

#### 2) Weekday Subset (WD)

The weekday subset calculates the features over only the data recorded from Monday to Friday.

#### 3) Weekend Subset (WE)

Conversely, the weekend subset calculates the features only over Saturday and Sunday.

#### 4) Median Subset

The median subset calculates the features over the following subsets of the data, and then, calculates the median value over these feature values: 1) the base, weekend, and weekday subsets described previously; and 2) the full week with each of the 7 days removed in turn.

#### 5) Optimized Daily Exclusion Subset

Some features such as the diurnal movement and location variance are particularly sensitive to outlier days where the individual travels much further than on other days. The optimized daily exclusion subset was developed to exclude days where the individual travelled a greater distance from home than usual.

The general principle is that the maximum Euclidean distance reached from home, *d_i_*, is calculated for each each day, *i* = 1*,* 2*, …,* 7, in set *D* = {*d*_1_, *d*_2_, …, *d*_7_}. The standard deviation *σ_D_* = SD (*D*) is calculated and any days where

(5) $$d_{i}>\text{median}\left(D\right)+\alpha \sigma_{D}$$

are removed. The value of *α* was optimized per feature to maximize the classification accuracy using a logistic regression classifier, which was found to provide the most accurate and consistent classification results in a single-feature setting.

### F Feature Extraction Summary

A total of 50 features (ten features for each of the five data subsets) were extracted from all 310 weeks of data (124 HC; 186 BD) with no missing values. As described earlier, analysis was only performed on the BD participants.

### G QIDS Score Estimation and Depression Classification

Mental health questionnaires such as QIDS or PHQ-9 are commonly used as tools to assess patient depression, and objective metrics using behavioral data could provide an additional clinical tool. This could be through estimation of the questionnaire score or by providing an indication of whether the patient is depressed or not.

Questionnaire responses were estimated from the features described previously using two models. The first model is a standard linear regression model [40] of the form

(6) $${\hat{Q}}_{i}=\beta_{0}+\beta_{1}f$$

where *Q̂*_*i*_ is the estimated QIDS score for participant *i*, *f* is a feature value, *β*_1_ is the feature weight and *β*_0_ is the intercept.

The second model is a generalized linear model (GLM) [40] with an underlying quadratic model and logistic link function

(7) $${\hat{Q}}_{i}=\frac{a}{1+e^{-\left(\beta_{0}+\beta_{1}f+\beta_{2}f^{2}\right)}}$$

which limits the range of *Q̂*_*i*_ smoothly between 0 and *a*. The GLM has the advantage of limiting the output values and allows greater complexity in the model, at the risk of overfitting the training data.

Classification of whether the participant is depressed (i.e., reports a QIDS score ≥ 11) was performed on the features described previously using a quadratic discriminant analysis (QDA) model, which models the data from each class as a multivariate normal distribution. The distributions from each class are allowed to have different covariances, which results in a quadratic decision boundary between classes (where the probability density functions of the two classes cross).

#### 1) Model Validation

Leave-one-participant-out cross-validation was used to validate the models, where all the labeled weeks for a participant were used for testing while the model was trained on the labeled weeks for the remaining participants. This was repeated for all participants to give an overall result. For depression detection, 10-fold and 5-fold cross-validation were also used to validate the models where the labeled weeks from 10% and 20% of participants were used for testing while the model was trained on the labeled weeks for the remaining participants. Finally, 3-fold cross-validation was used for depression detection, but instead of leaving whole participants out, the data for each participant was split into the three partitions and cross-validation performed like usual, hence allowing data for a participant to be used for both training and testing.

#### 2) Group Equalization

For depression detection, where the classes being classified were not equally sized, the training data were adjusted by subsampling the overrepresented class so that the results were not biased by the class proportions. This was applied to all cross-validation methods.

In each iteration of cross-validation, the training data had *n*_1_ samples from group *G*_1_, and *n*_2_ samples from group *G*_2_. In the case where *n*_1_ ≠ *n*_2_, *n*_min_ = min(*n*_1_, *n*_2_) samples were chosen from both *G*_1_ and *G*_2_ to form *G_T_* (i.e., all samples from the under-represented group were used, and the same number of samples were randomly chosen from the over-represented group). *G_T_* was used as the training data to train the model and performance was tested on the left-out weeks. The selection of *G_T_* was repeated *M* times for each fold or left out participant. Where *k*-fold cross-validation was used, the random generation of the *k* partitions was also repeated [*N/k*] times where *N* is the number of participants.

#### 3) Model Evaluation

The regression models for questionnaire score estimation are evaluated using the mean absolute error (MAE) from the true QIDS score *Q_i_* calculated by

(8) $$\text{MAE}=\frac{1}{N}\sum_{i=1}^{N}\left|Q_{i}-{\hat{Q}}_{i}\right|.$$

The results from the binary classification of depression (QIDS score ≥ 11 versus QIDS score < 11) are presented as accuracy (Ac), sensitivity (Se), specificity (Sp), and F_1_ score (F_1_). The F_1_ score is defined as

(9) $${\text{F}}_{1}=\frac{2\cdot \text{Se}\cdot \text{Sp}}{\text{Se}+\text{Sp}}$$

which combines the sensitivity and specificity with equal weighting to provide a single score [41].

Classification performance is also presented using the receiver operating characteristic (ROC) curve, which is defined as a plot of the false positive rate (FPR) against the true positive rate (TPR) of the classifier. The different values of FPR and TPR between 0 and 1 are generated by adjusting the class acceptance threshold for the classifier. The area under this curve (the AUC) is also presented as an indicator of the performance of the model to different inputs.

### H Feature Selection Using a Wrapper

Most regression and classification models are sensitive to the combination of features presented to them. Feature selection can maximize model performance and improve interpretability by choosing an optimal combination of features from the whole feature set.

Feature selection *wrapper methods* use the final classifier or regression model as part of the feature selection process, which means that they can make use of the particular properties of the classifier being used, as well as subtle properties of the individual features that may be missed with more general methods. A simple wrapper method was created that incrementally selects features based on the performance of each feature individually, then each remaining feature with the first selected feature etc. Regression feature selection was performed using the two regression models with leave-one-participant-out cross-validation. Features were selected using the MAE of the QIDS score estimation. Classification feature selection was performed using the QDA classifier with leave-one-participant-out or *k*-fold cross-validation with group equalization. Features were selected using the median F_1_ score of the classification result.

## III Results

### A QIDS Score Estimation

**Fig. 3:**
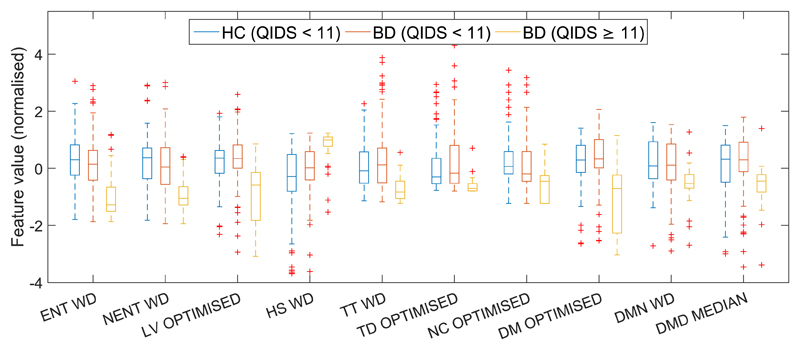
Feature distributions for features calculated on data subsets with optimal individual classification results. Feature abbreviations are given in Table II; WD: Weekday data subset; OPTIMIZED: Optimized daily exclusion data subset; MEDIAN: Median data subset.

All features were calculated on each of the five data subsets from the recorded data as described previously. Depression classification was performed on each feature individually on the BD participants and the feature distribution statistics for each feature from the data subset with the highest median F_1_ score are shown in Fig. 3. This shows BD participants with depressive symptoms (QIDS score ≥ 11), BD participants without depressive symptoms (QIDS score < 11) and HC participants, none of whom exhibit depressive symptoms. This demonstrates that there are clear differences in most of the features between BD participants with depressive symptoms and those without. It also shows that BD participants without depressive symptoms have feature distributions very similar to the HC participants, which suggests that while it is possible to distinguish depression, it is not possible to distinguish non-depressed BD from HC participants.

**Fig. 4:**
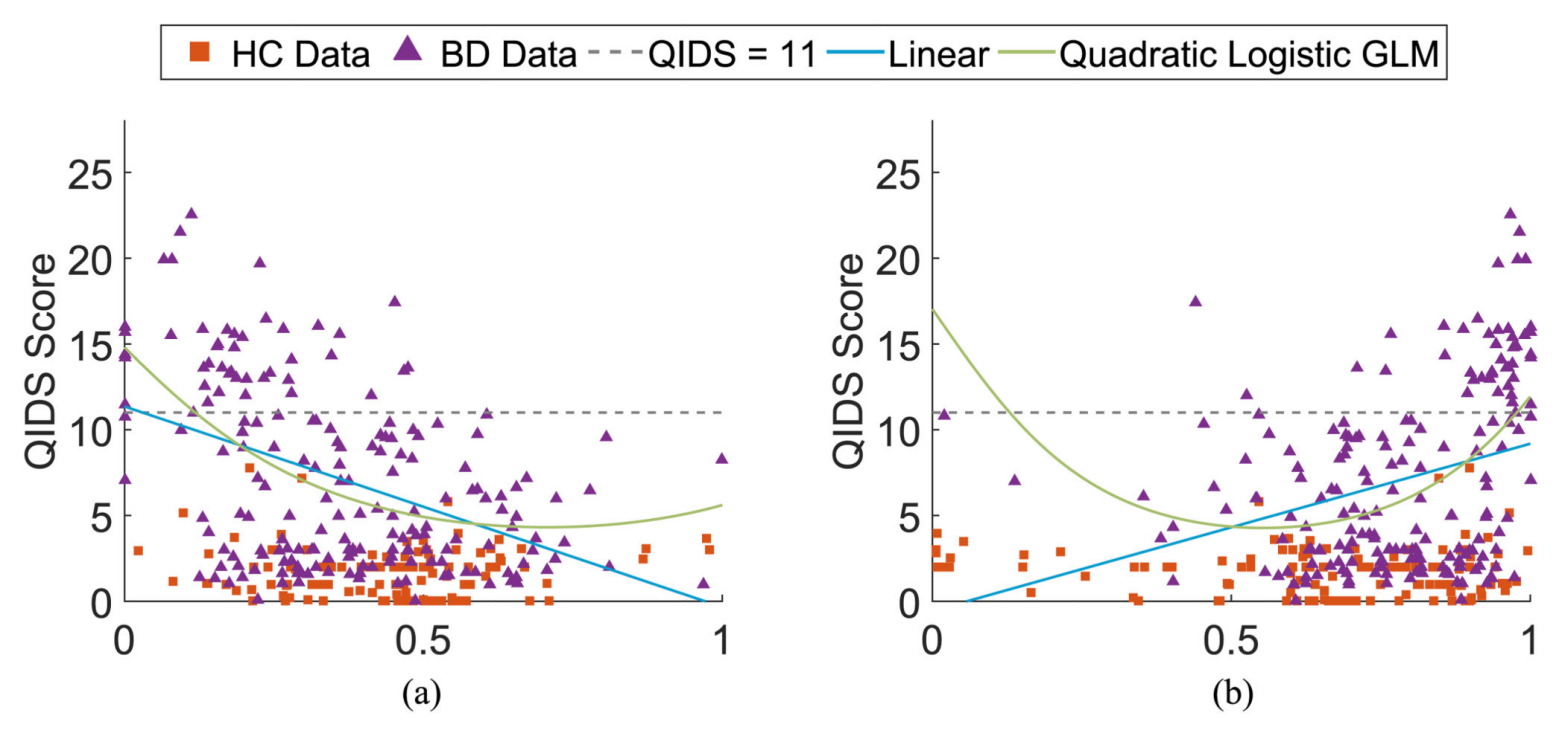
Features extracted from the weekday geolocation data from HC and BD participants, showing (a) normalized entropy; and (b) home stay. The standard linear regression model in (6) and GLM with a quadratic model and logistic link function in (7) calculated on the BP participants only are shown overlaid on each of the features. The dashed line shows the moderate depression threshold where the QIDS score is 11.

To explore this in greater detail, the relationship between the QIDS score and normalized entropy and home stay features calculated on the weekday data subset are shown in Fig. 4(a) and (b), respectively. The standard linear regression model (6) and the GLM with quadratic model and logistic link function (7) were both calculated on each feature for the BD participants and are shown overlaid. Both of these models demonstrate a correlation with the QIDS score, and the differences in feature values recorded above and below the cut-off for moderate depression are notable. Due to an insufficient range of QIDS scores reported by the HC participants, it is not possible determine if they follow the same model as the BD participants.

The error rates from the two regression models fitted to each feature on the BD participants are shown in Table III evaluated using the MAE (8). This shows the error rate for each feature calculated on the optimal data subset, i.e., the one with the lowest MAE for that feature and model. The MAE from the baseline constant-value model of the mean value of all the recorded QIDS scores is also shown. Combined results show the first *n* features selected by the wrapper feature selection method for each model that resulted in the optimal MAE. The significance level was evaluated using an F-test of the model against the baseline constant-value model, which evaluates the increase in performance against the increase in complexity of the model. The results in Table III are shown from leave-one-participant-out cross-validation and presented results are from all the left out weeks over all iterations. The lowest MAE using the standard linear model is 4.21 with the normalized entropy feature on the optimized daily removal data subset. The lowest MAE using the quadratic logistic GLM is 3.97 with the entropy feature on the weekday data subset. Both of these results are significant against the baseline constant-value model at a *p* < 0.0001 significance level. Combining features selected using the wrapper feature selection method resulted in the lowest MAE of 3.75 (*p* < 0.0001) for the standard linear model and 3.74 (*p* = 0.007) for the quadratic logistic GLM using the first 10 and 14 selected features, respectively. Given that the QIDS score ranges between 0 and 27, this demonstrates that regression on any of the features does not provide a good QIDS score prediction.

### B Depression Classification

**Fig. 5:**
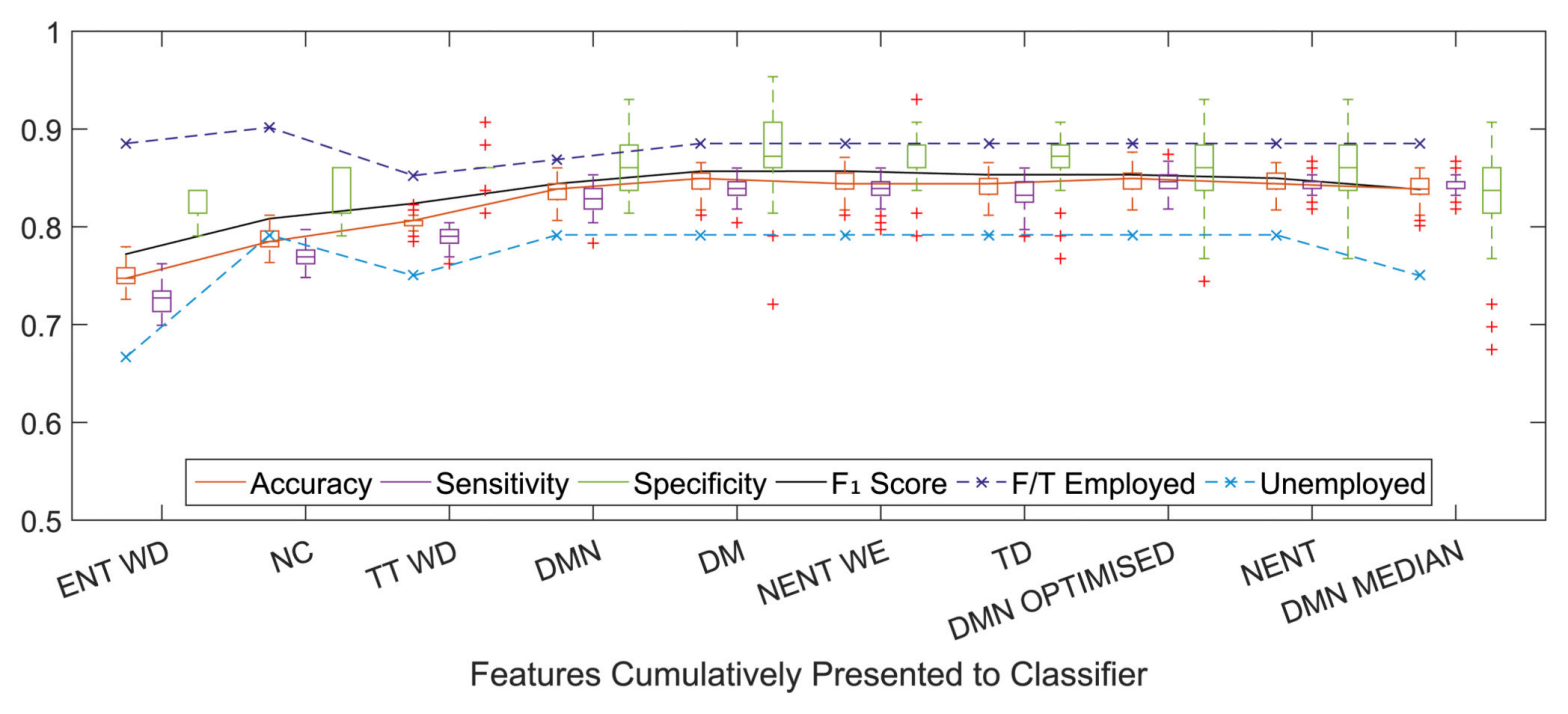
Classification results for depression detection model (based on labeled QIDS score ≥ 11) trained on BD participants only. Classification was performed using QDA with the leave-one-participant-out with group equalization method with 100 folds of group equalization for each left out participant. Features were presented to the classifier in the order selected by the feature selection wrapper method. Results are summarized in the form of box plots showing the median and interquartile range with outliers denoted with crosses. The classification accuracy for only the full-time employed and unemployed participants is also shown in the dashed traces. Feature abbreviations are given in Table II; WD: weekday data subset; WE: weekend data subset.

Results from classification of depression in the BD participants based on labeled QIDS score ≥ 11 are shown in Fig. 5 for the first ten features selected. The model was trained using leave-one-participant-out cross-validation and group equalization as described previously.

**Fig. 6:**
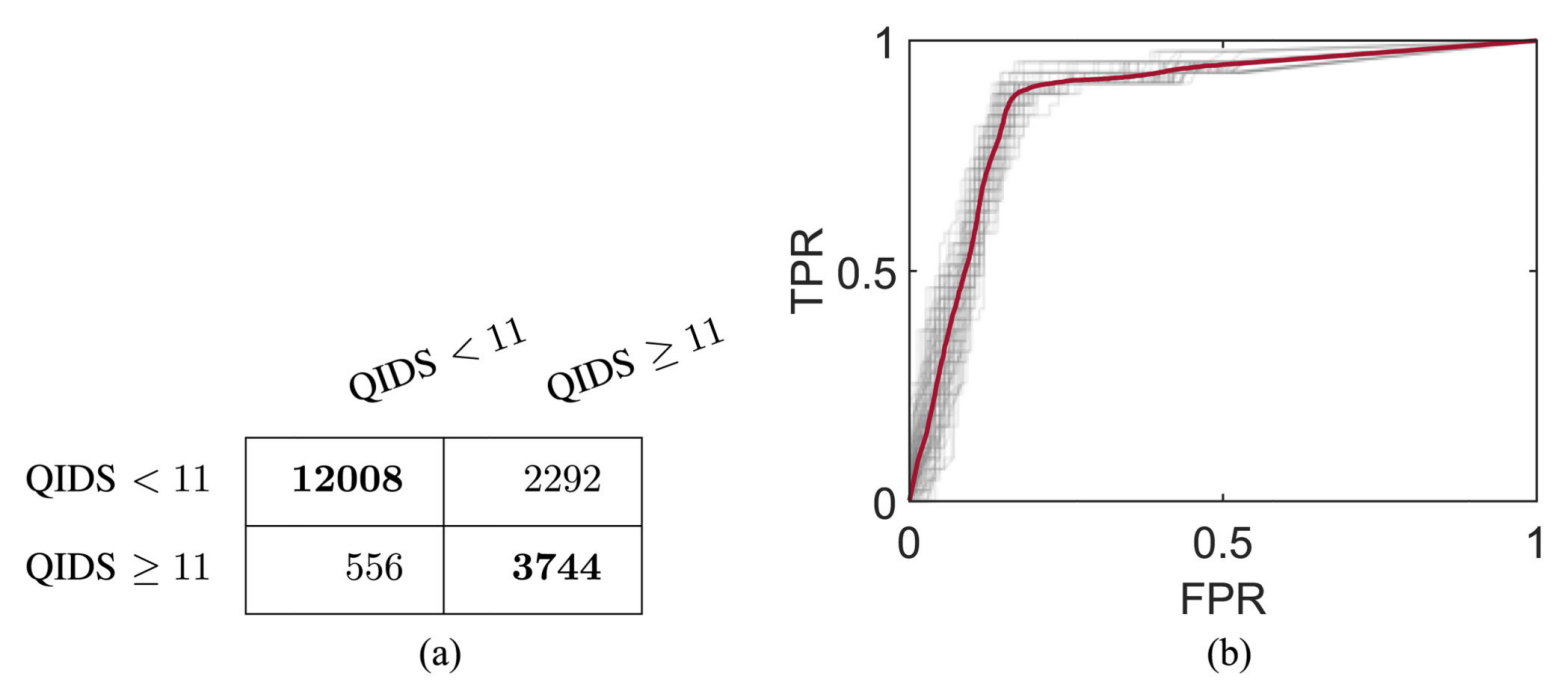
Performance metrics of the leave-one-participant-out classifier trained with the five features providing optimal classification accuracy. The confusion matrix (a) shows the classification of test samples of each class. Each row is the true class, and each column is the classification. The ROC graph (b) shows how the classifier performs on positive and negative test samples as the classification threshold is adjusted. The gray traces are the results from the individual models trained in cross-validation and the thicker red trace is the mean value. FPR: false positive rate; TPR: true positive rate.

The highest F_1_ score is achieved with five features where the accuracy (median ± IQR) is 0.849 ± 0.016 (Se: 0.839 ± 0.014; Sp: 0.872 ± 0.047). The confusion matrix for the test data in this optimal model with five features is shown in Fig. 6(a) and the ROC curve is shown in Fig. 6(b). The ROC curve demonstrates that it is possible to reach approximately 80% TPR rate with minimal false positives.

Full results from leave-one-participant out, 10-fold and 5-fold cross-validation are shown in Table IV. The robustness of the model is demonstrated by the close match in the accuracy from all three methods of cross-validation. The accuracy from the 3-fold cross-validation where the data from each participant is split into three groups for training and testing is also shown. Unsurprisingly the results from the 3-fold cross-validation improve on the other methods because the data for each participant is not independent over time.

To explore the potential effects of employment status on the results, Fig. 5 also shows the classification accuracy of only the full-time employed and unemployed BD participants. The classification of employed participants is generally better than unemployed participants, which is notable given the reliance on features indicating the level of regularity in routine and levels of geographic activity seen from participants.

## IV Discussion

In this study, we have demonstrated that it is possible to detect depressive episodes in individuals with bipolar disorder with 85% accuracy using geographic location recordings alone. While classification based on a single feature was not sufficient for maximal accuracy, the high correlation between features meant that maximum classification performance was achieved with five features. The choice of classifier was also found to be important with initial experiments using logistic regression performing much worse than the presented results using QDA. Objective features were, however, not accurate in providing an estimate of the total QIDS score.

These findings are consistent with those previously reported [21] and extend them to a more generalizable clinical sample in a community study. This demonstrates that geographic location movements can provide a useful metric for the identification of depressive symptoms in bipolar patients in community settings.

The misclassifications are most likely to have occurred because the nature of the relationship between mood and movement data may differ between individuals. In other words, not everyone will experience depression in the same way. For example, while staying at home and visiting fewer locations were both shown to be strong indicators of depression (see Fig. 3), someone in full-time employment may be constrained to a routine, even when unwell. Conversely, some people will have nonpathological reasons for choosing to stay at home, or not visit many locations.

Additionally, the causality between the mood and the objective measures is unclear. For example, a decline in movement may result from the presence of depressive symptoms but given the positive effects of exercise and social interaction/occupation upon mood the reverse relationship may also be present (or both). Whether this would impact classification is unknown, but there is a limit to how effective population-level models (as presented here) can be. To improve the results further, personalized modeling of features is likely to be required, as demonstrated by the improvement in performance seen when using 3-fold cross-validation, where the training data may come from the same participant as the test data.

Although this study has produced promising results, a number of other limitations have been identified as follows.

1) The performance of the method described is highly dependent on the data quality, which was found to be unpredictable on the Android platform used. The preprocessing methods developed were therefore crucial for extracting useful information about the behavior of the individual from the data available. Data collection in this study was relatively well controlled given that in most cases participants were provided with specific smartphones. Using different phones in a real-world setting might exacerbate this problem, however it was observed that newer models with increased processing capabilities generally provided higher quality data and so data issues are likely to become less significant in the future.
2) Geolocation recorded from smartphones only provides data as to the location of the handset and not necessarily its owner. There may have been times when the phone was not carried or was lent to someone else, however, given the widespread reliance on mobile technologies for daily life, the impact of this upon our findings is unlikely to be significant.
3) Weekday and weekend patterns were used as proxies for working and nonworking days, which in many cases is likely to not be entirely accurate. The effect of this on the results is unclear, but again more personalized methods may help.
4) Self-reporting of mood symptoms also has a number of limitations given the inherent bias in retrospective recall of mood states [42], however QIDS is a well validated and widely used measure for assessing mood in bipolar disorder.
5) Finally, only seven participants in the studied cohort exhibited depressive symptoms, and therefore, these results need to further validated on a larger scale.

## V Conclusion

To our knowledge, this is the first study of the utility of using geolocation data to detect depressive symptoms in a community sample of bipolar patients. Our findings suggest that features of geolocation may be a useful proxy for mood states. They need to be extended to explore mood changes within individuals but may prove to be useful tools in the early identification of depressive episodes and in guiding self-management.

## Figures and Tables

**Table I:** Demographic Data for Participants Who Provided Sufficient Data for Analysis

	HC	BD With Only QIDS Score < 11	BD With QIDS Score ≥ 11
Participants	14	15	7
Gender	Two male; 12 female	Five male; ten female	Two male; five female
Age (mean ± sd)	42 ± 14	46 ± 14	41 ± 15
BMI (mean ± sd)	24.5 ± 4.6	26.4 ± 3.4	29.3 ± 1.9
Employment status	Six full-time employed;		
	three part-time employed;	Six full-time employed;	
	three unemployed;	six part-time employed;	Two full-time employed;
	one student; and	two students; and	two part-time employed; and
	one retired	one unknown	three unemployed
Weeks of data (mean ± sd)	8.9 ± 3.2	8.4 ± 2.8	8.6 ± 3.6

**Table II:** Feature Overview

Feature Name	Abbr.	Description
Entropy	ENT	A measure of the variability in the time that participants spend in the different locations recorded, defined as $\text{ENT}=-\sum_{i=1}^{N}p_{i}\;\text{log}\;\;p_{i}$ where *i* = 1, …, *N* is the index of each location cluster extracted, *N* is the total number of clusters and *p_i_* is the percentage of time recorded cluster *i*.
Normalized Entropy	NENT	A variant of the ENT feature scaled to be in the range [0, 1], defined as NENT=ENT/logN.
Location Variance	LV	An indication of how much the individual is moving between different locations based on the sum of statistical variances in the latitude and longitude, defined as $\text{LV}=\text{log}\;\left(\sigma_{\text{lat}}^{2}+\sigma_{\text{lon}}^{2}\right).$
Home Stay	HS	The percentage of time that the participant is recorded in their home location.
Transition Time	TT	The percentage of all the time spent travelling between stationary locations in the data recorded.
Total Distance	TD	The sum of Euclidean distances between the consecutive location points recorded in the data, calculated as $\text{TD}=\sum_{i=2}^{N}\sqrt{{\left(x_{i}-x_{i}-1\right)}^{2}+{\left(y_{i}-y_{i}-1\right)}^{2}}$ where *i* = 1, …, *N* is the index of each of the total *N* pre-processed data points for the participant, and *x_i_* and *y_i_* are the distance east and north respectively from the assumed home location at coordinates (0, 0).
Number of Clusters	NC	The number of distinct location clusters extracted in the week-long data sections using the *K* -means method.
Diurnal Movement	DM	A measure of daily regularity quantified using the Lomb–Scargle periodogram to determine the power in frequencies with wavelengths around 24 h. The power spectral density (PSD) of the signal in selected frequencies with wavelengths between 23.5 h and 24.5 h is calculated and averaged as $E=\sum_{i=1}^{N}\text{psd}(f_{i})/N$ where psd (*f*) is the PSD of the data at frequency *f* and *f_i_* for *i* = 1, …, *N* is the range of frequencies to use with wavelengths between 23.5 h and 24.5 h. This gives a measure of the energy in the spectrum with wavelengths around 24 h. The diurnal movement feature is defined as $\text{DM}=\text{log}\;\;(E_{\text{lat}}+E_{\text{Ion}})$ where *E*_lat_ and *E*_lon_ are the energy values for the latitude and longitude of the recorded data respectively.
Diurnal Movement on Normalized Coordinates	DMN	Similar to the DM feature but calculated on a normalized set of coordinates, where the latitude and longitude are both scaled to have zero mean and unit variance within the period being classified.
Diurnal Movement on the Distance From Home	DMD	Similar to the DM and DMN features but calculated using the Euclidean distance from home, rather than latitude and longitude, normalized to have zero mean and unit variance within the period being classified.

**Table III:** Regression Model Error Rates on Optimal Data Subset

Feature	Baseline	Linear Model		Quadratic Logistic GLM	
			
	MAE	Data Subset	MAE	Data Subset	MAE
ENT	4.724	Weekday	4.432***	Weekday	3.968***
NENT	4.724	Optimized	4.207***	Weekday	4.125***
LV	4.724	Optimized	4.276***	Optimized	4.205***
HS	4.724	Weekday	4.602***	Weekday	4.197***
TT	4.724	Weekend	4.530***	Weekend	4.520**
TD	4.724	Base	4.664**	Median	4.666
NC	4.724	Weekend	4.516**	Weekend	4.576*
DM	4.724	Optimized	4.262***	Optimized	4.215***
DMN	4.724	Optimized	4.629**	Optimized	4.618*
DMD	4.724	Optimized	4.491***	Optimized	4.503***
Combined	4.724	Ten Features	3.748***	14 Features	3.737**

Significance of fitted model from the baseline model indicated by asterisks: * < 0.05; ** < 0.01; *** < 0.001. Feature abbreviations are given in Table II; MAE: mean absolute error.

**Table IV:** Depression Classification Results

No. Features	LOO Cross-Validation		10-fold Cross-Validation		5-fold Cross-Validation		3-fold Cross-Validation	
	Accuracy	AUC	Accuracy	AUC	Accuracy	AUC	Accuracy	AUC
1	0.747 ± 0.016	0.810 ± 0.014	0.747 ± 0.016	0.811 ± 0.017	0.753 ± 0.027	0.812 ± 0.028	0.758 ± 0.022	0.824 ± 0.011
2	0.785 ± 0.016	0.833 ± 0.016	0.780 ± 0.011	0.837 ± 0.016	0.780 ± 0.027	0.825 ± 0.026	0.785 ± 0.022	0.855 ± 0.010
3	0.806 ± 0.005	0.867 ± 0.012	0.801 ± 0.016	0.867 ± 0.014	0.801 ± 0.022	0.859 ± 0.021	0.817 ± 0.022	0.885 ± 0.012
4	0.839 ± 0.016	**0.882** ± 0.014	0.828 ± 0.016	0.871 ± 0.023	0.844 ± 0.022	0.878 ± 0.022	0.833 ± 0.016	0.900 ± 0.013
5	**0.849** ± 0.016	0.878 ± 0.013	0.833 ± 0.022	0.876 ± 0.016	**0.860** ± 0.022	**0.889** ± 0.015	0.876 ± 0.011	0.909 ± 0.010
6	0.844 ± 0.016	0.871 ± 0.017	**0.844** ± 0.016	**0.879** ± 0.016	0.849 ± 0.022	0.887 ± 0.024	0.887 ± 0.016	**0.919** ± 0.009
7	0.844 ± 0.016	0.869 ± 0.014	0.833 ± 0.016	0.869 ± 0.021	0.839 ± 0.054	0.885 ± 0.078	**0.892** ± 0.011	0.908 ± 0.012
8	**0.849** ± 0.016	0.879 ± 0.015	0.839 ± 0.016	0.869 ± 0.018	0.833 ± 0.027	0.882 ± 0.024	0.882 ± 0.022	0.908 ± 0.020
9	0.844 ± 0.016	0.867 ± 0.015	0.839 ± 0.016	0.872 ± 0.018	0.817 ± 0.027	0.855 ± 0.042	0.876 ± 0.016	0.899 ± 0.021

All values are presented as median ± IQR. Classification was performed using 100 iterations of group equalization for each left out participant or fold. 3-fold cross-validation was performed by splitting all the data for each participant into the three partitions to use for cross-validation.

## Footnotes

1 The AMoSS study was approved by the East of England NHR research ethics committee (REC reference 13/EE/0288) and all participants provided written informed consent.

2 The term “circadian” strictly refers to the internal 24 h biological clock that guides our daily routines based on environmental factors such as light and temperature [36]. Since it would not be possible to measure these biological processes using only geographic location data, for the remainder of this paper, the term “diurnal” has been used instead to indicate that we are only providing a measure of daily regularity, which may not be driven by the underlying circadian clock.
